# Local field potential decoding of the onset and intensity of acute pain in rats

**DOI:** 10.1038/s41598-018-26527-w

**Published:** 2018-05-29

**Authors:** Qiaosheng Zhang, Zhengdong Xiao, Conan Huang, Sile Hu, Prathamesh Kulkarni, Erik Martinez, Ai Phuong Tong, Arpan Garg, Haocheng Zhou, Zhe Chen, Jing Wang

**Affiliations:** 1 Department of Anesthesiology, Perioperative Care, and Pain Medicine, New York University School of Medicine, New York, 10016 USA; 2 College of Biomedical Engineering and Instrument Science, Zhejiang University, Hangzhou, 310027 China; 3 Department of Psychiatry, New York University School of Medicine, New York, 10016 USA; 4 Department of Neuroscience and Physiology, New York University School of Medicine, New York, 10016 USA

## Abstract

Pain is a complex sensory and affective experience. The current definition for pain relies on verbal reports in clinical settings and behavioral assays in animal models. These definitions can be subjective and do not take into consideration signals in the neural system. Local field potentials (LFPs) represent summed electrical currents from multiple neurons in a defined brain area. Although single neuronal spike activity has been shown to modulate the acute pain, it is not yet clear how ensemble activities in the form of LFPs can be used to decode the precise timing and intensity of pain. The anterior cingulate cortex (ACC) is known to play a role in the affective-aversive component of pain in human and animal studies. Few studies, however, have examined how neural activities in the ACC can be used to interpret or predict acute noxious inputs. Here, we recorded *in vivo* extracellular activity in the ACC from freely behaving rats after stimulus with non-noxious, low-intensity noxious, and high-intensity noxious stimuli, both in the absence and chronic pain. Using a supervised machine learning classifier with selected LFP features, we predicted the intensity and the onset of acute nociceptive signals with high degree of precision. These results suggest the potential to use LFPs to decode acute pain.

## Introduction

Pain has sensory, affective and cognitive dimensions. Acute pain protects us from injury. Chronic pain, however, affects up to 30% of adults and represents a form of maladaptive condition. Currently, pain is diagnosed by patient report. In animal studies, it is defined by spinal withdrawal, or more recently by conditioned aversive behaviors^1^. There are limitations to these behavioral definitions for pain. In patients, verbal report may be inaccurate, especially in those patients who experience cognitive impairments. They are also problematic in children, the elderly or people with language barriers. In animal studies, neither spinal withdrawal nor conditioned aversion is completely specific for pain. Thus, finding a reliable and objective diagnosis for pain remains one of the key challenges in sensory neuroscience.

Previous neuroimaging studies have identified brain-wide circuit changes that correspond with acute pain^2–6^. One region that has been shown to correlate with the affective processing of pain is the anterior cingulate cortex (ACC). Studies in humans have suggested that the ACC may even provide evaluation for the intensity of acute pain^7,8^. Meanwhile, studies in animal models have demonstrated that ACC is both necessary and sufficient for the acquisition of aversive learning in acute and chronic pain conditions^9–13^. Furthermore, a limited number of studies using *in vivo* recordings in freely moving rats have shown that individual neurons in the ACC may carry information for both the intensity and onset of pain^13,14^.

The acquisition for extracellular spike trains of individual neurons requires invasive recordings. Stable chronic recordings of individual neurons in rodents or primates remain a challenging technique, and hence are not well suited for studies on chronic pain. In contrast to spikes that represent single unit outputs, local field potentials (LFPs) are comprised of a combination of synaptic and network activities within a local brain region and are relatively stable across time domain. LFPs are thought to represent the aggregate subthreshold activity of neurons in a localized area near the recording electrode, and can be viewed as the input information in that specific local brain area^15^. Since LFPs measure the collective behavior of ensembles of neurons, frequency-specific LFPs are thought to process distinct network information. For example, theta oscillation (4–8 Hz) in the hippocampus has been shown to be important for arousal and the formation of spatial memory in rodents^16,17^. Meanwhile, high frequency cortical gamma oscillation (30–100 Hz) is known to be important for sensory processing, as the high-frequency LFP power can provide a proxy for the assessment of neuronal outputs^15^. Both theta and gamma band activities have recently been shown to play a role in pain perception^18–24^. However, to date no study has examined the specificity of LFPs in predicting either the onset or the intensity of acute pain, and how such decoding is altered by the presence of chronic pain. The ability to interpret LFPs also has direct implications to clinical neuroscience, since LFPs bear some similarities to electroencephalogram (EEG) signals that are routinely recorded non-invasively^25^. Here we recorded LFPs from the ACC of freely behaving rats, and used a machine-learning classifier with extracted LFP features to decode the onset and intensity of nociceptive signals. We found that our method provides relatively high specificity and sensitivity for these signals, even in chronic pain conditions, suggesting the potential for LFPs in the decoding of acute pain.

## Results

### LFPs in the ACC demonstrate changes in response to peripheral noxious stimulations

**Figure 1:**
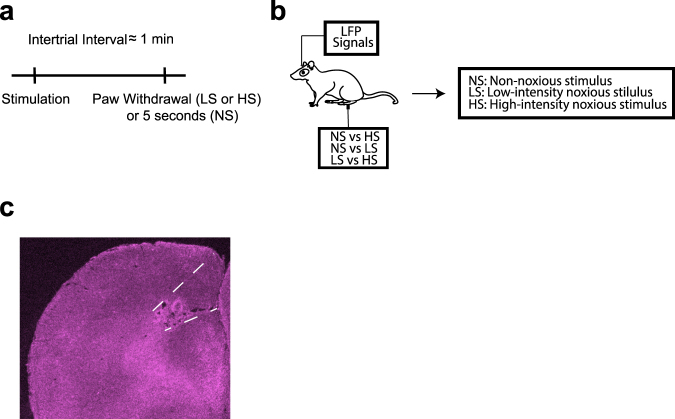
Experimental paradigm. (**a**) Timeline and schematic for electrophysiological recordings in freely moving rats. Each trial of peripheral stimulation lasted until paw withdrawal or in cases of no withdrawal (non-noxious stimulus or NS) a total of 5 s. (**b**) Local field potentials (LFPs) were recorded from the anterior cingulate cortex (ACC) contralateral to peripheral stimulations. Stimulations were done with non-noxious thermal stimulus (NS), low-intensity noxious stimulus (LS), or high-intensity noxious stimulus (HS), through adjustment of power outputs from a laser. (**c**) Histological images showing the location of tetrode recordings in the ACC.

We used chronically implanted tetrodes to record extracellular activities (spikes and LFPs) from the ACC of freely moving Sprague-Dawley rats (Materials and Methods). We recorded LFPs before, during and after peripheral stimulations (Fig. 1a). Stimulations were done with a laser directed at the hind paw of rats through a mesh table (Fig. 1b). By adjusting the power of the laser output, we could adjust the heat applied to the hind paw, and stimuli were classified as non-noxious (NS), low-intensity noxious (LS), and high-intensity noxious stimuli (HS)^13,14^.

**Figure 2:**
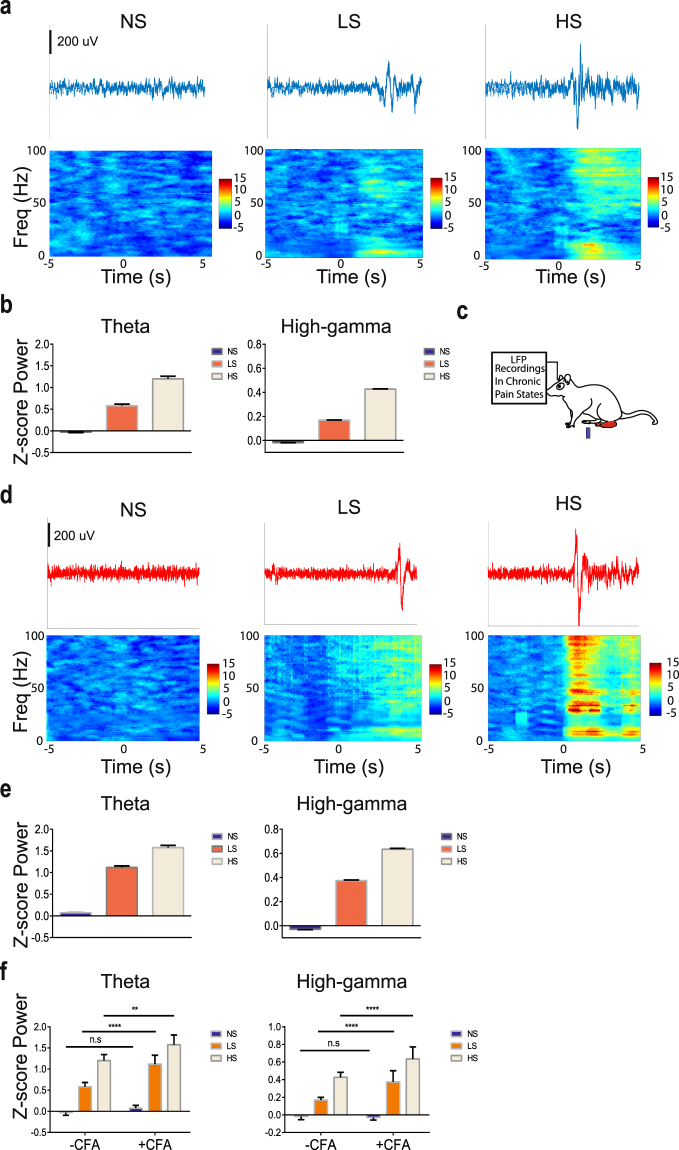
LFPs in the ACC provide information on the intensity of acute thermal pain. (**a**) Examples of LFP raw traces. Time 0 denotes the onset of laser stimulation (from left to right: NS, LS, HS). Each panel below the single-trial LFP traces shows trial-averaged event-triggered time-frequency spectrum. Time 0 denotes the onset of laser stimulation (from left to right: NS, LS, HS). (**b**) The Z-score power in two frequency bands demonstrates increased power with noxious stimulations (from left to right theta (4–8 Hz) and high-gamma (60–100 Hz) bands). (**c**) Schematic for CFA treatment to induce chronic pain. CFA was injected in the paw that is ipsilateral to ACC implants. Peripheral stimulations were performed in the contralateral paw to ensure consistency in recording from the contralateral ACC. (**d**) Examples of single-trial LFP raw traces in CFA-treated rats. Time 0 denotes the onset of laser stimulation (from left to right: NS, LS, HS). Each panel below the LFP traces shows trial-averaged event-triggered time-frequency spectrum after chronic pain. Time 0 denotes the onset of laser stimulation (from left to right: NS, LS, HS). (**e**) The Z-score power in two frequency bands demonstrates increased power with noxious stimulations in CFA-treated rats (from left to right theta (4–8 Hz) and high-gamma (60–100 Hz) bands). (**f**) Chronic pain induces increased power in theta and high gamma bands. **P < 0.01; ****P < 0.0001 two-way ANOVA.

Multi-channel LFPs were recorded from the ACC that was contralateral to peripheral stimulations. The trial-averaged LFP spectrogram shows that noxious stimulations with LS or HS increased the power at theta and gamma frequencies (Fig. 2a). As previous research has indicated the importance of theta and high gamma oscillations in sensory cortices for pain perception^18–24^, we quantitatively compared the power in theta and gamma bands with NS, LS or HS (Fig. 2b). The enhancement of theta and high gamma frequency bands in the spectrogram reflects the so-called event-related synchronization (ERS) phenomenon observed in freely moving rats^24^ and in human EEG/ECoG studies^23^. Together, these results show that as we increased the noxious intensity, there was an increase in power in both theta (4–8 Hz) and high gamma (60–100 Hz) bands in the ACC. Next, we analyzed if these increases in theta and high gamma powers were also seen in rats with chronic pain. For these experiments, we used Complete Freund’s Adjuvant (CFA) injection to induce pain in the limb that is ipsilateral to ACC tetrode implants, and then stimulated the opposite, uninjured paw (Fig. 2c). Therefore, we recorded LFPs in the ACC that was contralateral to peripheral stimulations. Similar to rats without chronic pain, CFA-treated rats demonstrated increased theta and gamma powers in response to LS or HS (Fig. 2d,e). Interestingly, however, compared with rats without chronic pain, CFA-treated rats showed a significant increase in power in the theta and high gamma ranges, particularly for LS stimulations (Fig. 2f). These results demonstrate, for the first time, that ensemble low frequency activities in the ACC are different in the chronic pain state.

### LFPs in the ACC encode information on the intensity of noxious stimulations

**Figure 3:**
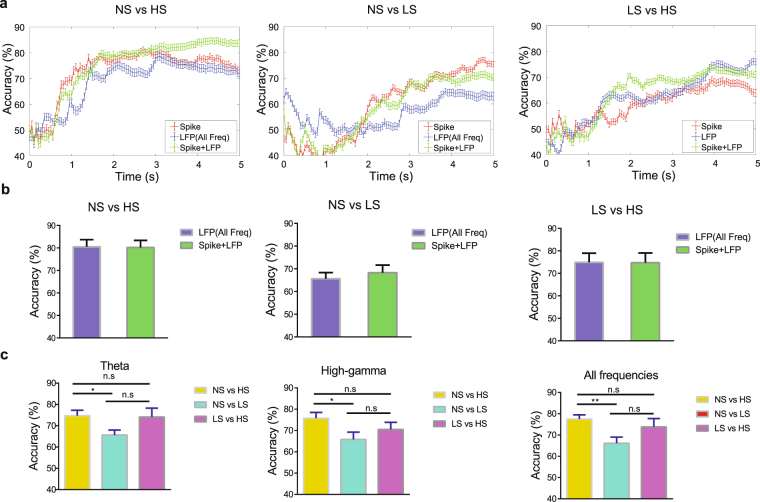
LFP decoding analysis using supervised machine learning predicts the intensity of pain. (**a**) Examples of recording sessions showing the comparison of detection accuracy with accumulated LFP (all frequency bands) and spike features. The detection accuracy was evaluated on the cross-validated testing data (see Materials and Methods). The error bar shows the S.E.M. from 100 Monte Carlo runs. (red: spike features alone, blue: LFP features alone, green: combined spike and LFP features). (**b**) Group comparisons of decoding peak accuracy between LFP features alone, and spike and LFP features together. (**c**) Accuracy to distinguish between HS and NS, LS and NS, or HS and LS with LFP features from different frequency bands (from left to right: theta, high-gamma, all frequency). *P < 0.05, rank-sum test.

We have previously shown that ensemble spike information in the ACC can be used to decode the intensity of pain^13^. Our results here suggest that LFPs can also be utilized for intensity decoding. In order to make an unbiased assessment of the contribution of LFPs in the ACC for pain intensity decoding, we used a support vector machine (SVM) classifier (see Materials and Methods). Specifically, we analyzed broadband power spectra (4–100 Hz, “all frequency” except for very low-frequency (1–3 Hz) bands subject to artifacts) based on multiple LFP recording sessions, each session containing at least 30 trials. During each session, we stimulated the rats with equal numbers of two different stimuli (NS vs HS, NS vs LS, or LS vs HS). In cross-validated LFP decoding analysis, we used 80% subsets of the trials for training the SVM classifier, and the remaining trials for testing the accuracy for the classifier to predict the correct identify of the stimulus. Our decoding analysis yielded high accuracy in distinguishing between NS and HS (~80% cross-validated accuracy), and reasonable accuracy in distinguishing between NS and LS (~65%), or between LS and HS (representative examples in Fig. 3a). Interestingly, when we compared the decoding accuracy using LFP features alone with the accuracy using spikes of sorted units from the same recordings^13^ (see Materials and Methods), the peak decoding accuracies for LFPs were slightly better. Combining spike and LFP features further improved the decoding accuracy curve, especially on the slope (Fig. 3a), but not on the peak accuracy (Fig. 3b). In some examples, when we combined spike and LFP features in decoding, we were able to achieve nearly 85% peak accuracy for distinguishing between NS and HS and the decoding accuracy arose quickly (<1 s) after the laser stimulation (Fig. 3a, left panel). Adding spikes to LFP features might not change the peak decoding accuracy (example in right panel of Fig. 3a and group statistics in Fig. 3b), but could change the timing or slope of decoding curve. Overall, this unbiased decoding analysis supported a critical role of ACC neurons in the representation of pain, and further demonstrated that LFP signals can be used to predict nociceptive intensity. This was also in line with the results on prediction of subjective pain perception based on human EEG recordings^25^.

Next, we examined the roles of specific theta and high gamma bands in pain intensity decoding. We applied the SVM classifier using the features computed from either theta or high gamma power. We found that these two frequency bands were almost as informative as the whole LFP range to achieve a similar accuracy level in decoding pain intensities (Fig. 3c). We observed a higher decoding accuracy in NS vs. HS (~75%) than in NS vs. LS (~65%), for both theta and high gamma frequency bands (P < 0.05, rank sum test).

### LFPs in the ACC encode information on the intensity of noxious stimulations in the chronic pain state as well

**Figure 4:**
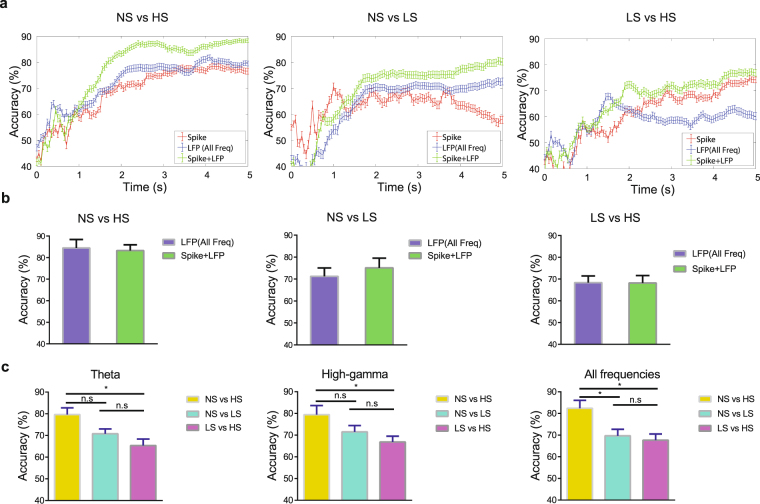
Chronic pain alters the decoding accuracy for noxious intensity. (**a**) Examples of recording sessions showing the comparison of detection accuracy with accumulated LFP and spike features after chronic pain in CFA-treated rats. The detection accuracy was evaluated on the cross-validated testing data. The error bar shows the S.E.M. from 100 Monte Carlo runs. (red: spike features alone, blue: LFP features alone, green: combined spike and LFP features). (**b**) Group comparison of decoding peak accuracy between LFP features alone, and spike and LFP features together in CFA-treated rats. (**c**) Accuracy to distinguish between HS and NS or LS and NS or HS and LS with LFP features from different frequency bands after chronic pain (from left to right: theta, high-gamma, all frequency). *P < 0.05, rank-sum test.

Epidemiological studies have shown that the presence of chronic pain can lead to increased perception of acute pain intensity and a distortion of pain intensity scale in a diffuse anatomic pattern^26–30^. Chronic pain is known to increase synaptic plasticity in the ACC^31,32^. We have shown previously that this enhanced plasticity in the ACC is anatomically nonspecific and is at least partially responsible for the generalized effect of chronic pain on acute pain perception^13^. Furthermore, this disruption in the ACC representation of pain can result in decreased decoding accuracy of pain intensity based on ensemble spike activity of individual neurons, suggestive of distortion of pain intensity scale found in chronic pain patients^13^. Here, we examined if this reduction in decoding accuracy is also found in LFP-based decoding analysis. We repeated decoding analyses in rats 10 days after CFA treatment (Fig. 4a,b). There was a modest decrease in the decoding accuracy (using the theta power feature) to distinguish between LS and HS (Fig. 4c; Supplementary Table 1). These results are compatible with our previous study using SVM based on spikes of sorted units^13^. Overall, however, it can be seen that LFPs can still be used to encode the intensity of noxious stimulation even in the chronic pain state.

### Population-decoding methods to detect the onset of acute pain

**Figure 5:**
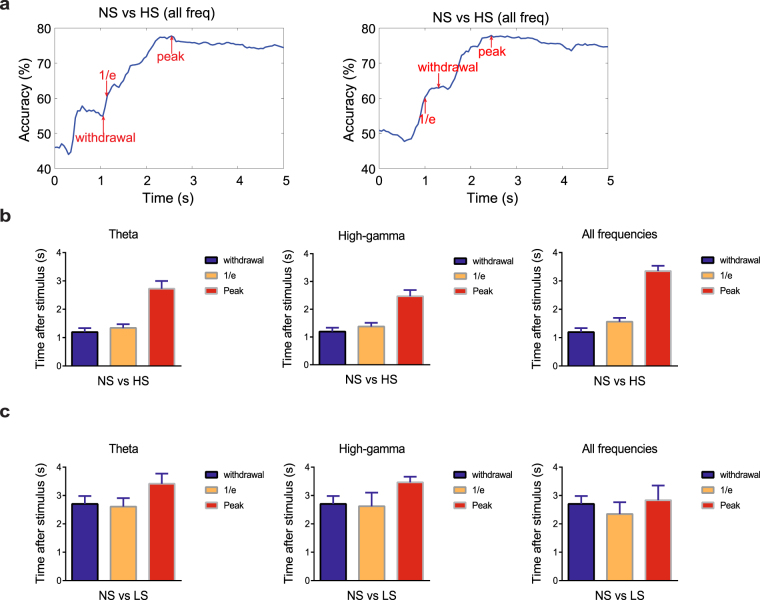
LFP decoding analyses can predict the onset of pain. (**a**) Two NS vs. HS examples of distinct detection accuracy trajectories based on supervised machine learning analysis using accumulated LFP features. The left and right examples show the withdrawal occurs around (before or after) the 1/*e* threshold for pain decoding. “Withdrawal” denotes the time of paw withdrawal after stimulus (time 0), “peak” denotes the onset of maximum accuracy for detecting the difference between NS and HS based on decoding analysis of LFPs in the ACC, and “1/*e*” denotes the time where the peak accuracy drops to the 1/*e* threshold above the baseline. (**b**) Assessment of the onset for pain perception in response to HS is based on the time where the decoding accuracy (NS vs HS) reached the 1/*e* threshold, using LFP features from different frequency bands (from left to right: theta, high-gamma, all frequency). This assessment of pain perception corresponds well with latency to withdrawal from the stimulus onset. (**c**) Same analysis as panel b, but for NS vs LS.

Detection of the onset of pain is a difficult task in freely behaving rodents for at least two reasons. First, single-trial neural signals are noisy and highly variable, at both single cell and population levels. Second, the number of pain-modulated neurons are relatively small (20–30%) in each recording session, limiting the signal-to-noise ratio (SNR) in signal detection. To address this problem, we used population-decoding analyses, based on the LFP alone, or combined LFP and spike information, to decode the onset of acute pain signals (Materials and Methods). As illustrated in Fig. 5a, we calculated the time where the peak decoding accuracy dropped to the 1/*e* threshold above the baseline (time 0). Interestingly, this 1/*e* time occurred either before or after the paw withdrawal within a reasonably narrow range (Fig. 5a). The rationale for using this 1/*e* time was motivated by the criterion for defining the visual neuronal receptive field size^33^. In addition, we have compared this criterion with an alternative criterion of using the ½ time to reach peak decoding accuracy (Supplementary Fig. 1). We used this moment as a proxy of the onset of the pain signals (“1/*e* onset”), which may occur before or after the paw withdrawal behavior. We then quantitatively compared the timing of the paw withdrawal, 1/*e* onset, and peak accuracy of decoding (based on different LFP frequency band features) between different laser intensities (NS vs HS, NS vs LS, LS vs HS; Fig. 5b,c, Supplementary Table 2, Supplementary Fig. 1). We found that the 1/*e* time indeed coincides very well with paw withdrawal time, and it was in fact comparable to the alternative criterion of ½ time. These results suggest that our machine learning algorithm may reveal important information about the onset of pain.

### Onset detection of acute pain is preserved in the chronic pain condition

**Figure 6:**
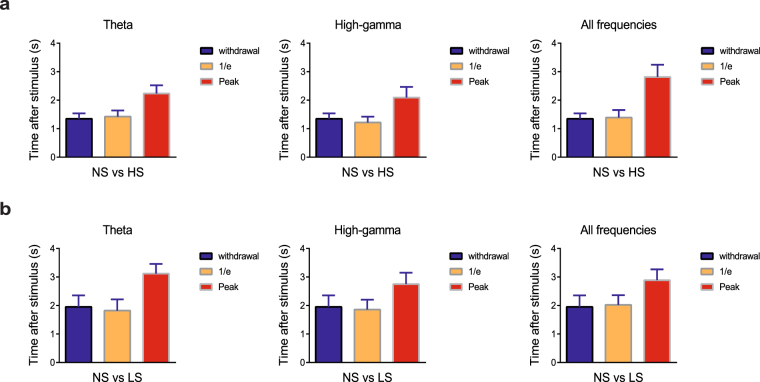
LFP decoding analyses can predict the onset of pain in CFA-treated rats. (**a**) Assessment of the onset for pain perception in response to HS is based on the time where the decoding accuracy (NS vs HS) reached the 1/*e* threshold, using LFP features from different frequency bands (from left to right: theta, high-gamma, all frequency). This assessment of acute pain perception corresponds well with latency to withdrawal from the stimulus onset in CFA-treated rats. (**b**) Same analysis as panel a, but for NS vs LS.

Next, we investigated the usefulness of the 1/*e* criteria in detecting the onset of pain in rats that experience chronic pain. In CFA-treated animals (Fig. 6a,b), we observed similar correlation between paw withdrawal time and 1/*e* time to peak detection of pain (Supplementary Table 2). These results indicate that our novel population-decoding method can be applied to detect the onset of acute pain even in rats with chronic pain.

## Discussion

In this study, using an unbiased neural decoding analysis, we have achieved a good decoding accuracy of pain intensity (especially for NS vs. HS) based on the LFP alone in the rat ACC, or based on a combination of LFP and spike information. Furthermore, we have shown that LFPs can also be used to assess the onset of acute nociceptive processing that correlates very well with spinal withdrawal behaviors.

Our analysis demonstrates the feasibility to use a combination of LFP and individual spike information to detect acute pain. Although the decoding accuracy for distinguishing NS vs. LS was lower compared with the decoding accuracy for NS vs. HS, increasing unit yield (e.g., ~40 units) can potentially increase the classification accuracy. Our results for NS vs. HS classification accuracy were also consistent with data from a generalized-linear model (GLM)-based decoding study using high-dimensional LFP features across multiple rat brain regions^34^. Meanwhile, to detect the onset of acute pain based on LFP, we have used the 1/*e* criterion. Most of our findings were robust with respect to an alternative criterion (e.g. an ½ criterion, Supplementary Fig. 1), as well. For instance, when comparing NS vs. HS, since the classification curve slope was very steep, the two criteria gave similar results. Meanwhile, when comparing NS vs. LS, the classification curve slope was less steep, and therefore there was a minor difference in the latency to detection between the two criteria. While we have tested these two criteria, there is actually no consensus or prior precedent in the literature to choose one method over the other. Therefore, depending on the choice of criteria, our decoding analysis will provide slightly different results. Nevertheless, these data demonstrate the potential for neural information in the ACC to decode both the intensity and the onset of acute nociceptive signals.

Since the LFP provides information about the collective behavior of neural ensembles, it can reveal both spectral and temporal scales of neural activity. For example, the high-frequency (gamma) LFP power can provide indirect assess to the spike outputs of neuronal ensembles^15^. In the literature, LFP and ensemble spike activity of the motor cortex have been demonstrated in decoding movement kinematics^35,36^. In contrast to the microscopic-level spiking and macroscopic-level EEG/ECoG data, high-frequency LFP power may serve as an intermediate, mesoscopic-level of neural data for reporting important brain signals. In this way, algorithms that explore LFP decoding capability can provide a stepping-stone to decoding using non-invasive recordings.

Theta and gamma oscillations have been shown to play important roles in previous studies on pain^18–24^. Theta oscillations are often associated with the hippocampus during locomotion, sleep, and memory formation, but it can also be detected in cortical and subcortical brain structures. Importantly, a recent study of freely moving rat EEG recordings has demonstrated theta ERS in response to nociceptive stimulations, indicating that brain oscillations in specific frequencies play an important role in acute pain states^24^. Pronounced increase of theta activity has also been observed in patients with chronic neurogenic pain^37^. Furthermore, human EEG recordings have shown that theta oscillations may contribute to the modulation of subjective pain sensitivity^22^. In addition, theta rhythmicity can also reflect a brain-state of social arousal in response to social and fearful stimuli, further contributing to the pain experience^38^. In human magnetoencephalography (MEG) studies, amplitude of gamma oscillations in the primary somatosensory cortex (S1) correlates with the “objective” stimulus intensity and “subjective” pain rating^20^. Another human EEG study has also shown that gamma-band oscillations in the contralateral S1 correlated with pain perception when stimuli are presented in isolation, but not when the saliency of such stimuli is reduced by repetition^21^. Furthermore, theta and beta oscillations of human LFP activities from sensory thalamus and periventricular gray/periaqueductal gray (PVAG) were shown to correlate inversely with pain relief induced by deep brain stimulation (DBS)^39^. Overall, results from these and other previous studies strongly suggest that both theta and gamma oscillations or theta- and gamma-ERS provide an important mechanism for the internal cortical representations and subsequent processing of peripheral nociceptive inputs^18–24,40,41^. LFP oscillations have been studied in several other acute pain models in awake rodents^42^. In a study of free-moving rats during carrageenan treatment, LFP activity in the ACC showed significant changes in a wide range of low frequency bands (1–30 Hz). Our findings are comparable to these results. At the same time, there were also divergent LFP findings observed in studies of other cortical regions. One study, for example, showed that a peripheral noxious stimulus (high-intensity transcutaneous electrical stimulation, mechanical pinch or formalin injection) induced a decrease in the LFP power (especially in theta and alpha bands) in the rat prelimbic PFC region. Interestingly, in contrast to the ACC, the prelimbic PFC has been shown to inhibit, rather than to process or enhance, pain phenotypes^43^. Thus, different cortical regions that have unique nociceptive-processing functions may also exhibit distinct LFP changes. In addition, as the perception of pain continues to be characterized by significant inter- and intra-subject variabilities in human and animal studies, an important challenge in this field is an effort to improve the use of LFP or EEG data to classify or predict the perception of pain across subjects.

In addition to the LFP power, the amplitude or latency of the pain-evoked potentials or laser-evoked potentials (LEPs) has been known to provide additional information for understanding the intensity of pain^21,44^. A key insight from our work is that unbiased assessment of LFPs demonstrates that these field potentials are as informative about the intensity of pain as pain-evoked spike events from single units or population. Furthermore, to our knowledge, this is the first study to utilize a decoding analysis approach to detect the onset of pain. This approach holds great promise as a tool to understand relevant neural circuitry for pain. For example, future studies can employ our method to examine LFPs, and possibly the coherence between LFPs or between LFPs and spikes across different relevant brain regions, to decode the onset of acute pain. The accuracy of onset decoding, and the timing of optimal decoding, can indicate the propagation of nociceptive information within defined neural circuits.

Single-trial decoding of the intensity or onset of pain remains a challenging yet important task. The reasons for such importance are two-fold. First, trial averaging can potentially underestimate valuable information in individual single trials. Second, single-trial spike/LFP analysis is very critical for detection/classification tasks during closed-loop brain-machine interface (BMI) experiments. Currently, single-trial decoding has been advanced to implement the development of closed-loop sensory or motor BMIs. A BMI directed treatment for pain, however, requires better understanding of neurofeedback^12,34^. Combining spikes and LFP, from single or multiple brain regions, will help advance this goal. Meanwhile, our proposed method was used for detecting transient acute pain signals in control and CFA-treated animals. How to extend the LFP-based method for detecting chronic pain signals should be the subject of future investigation.

Circuit mechanisms may differ among various types of pain. An important aspect of our work is that our decoding accuracy is preserved in the chronic inflammatory pain condition. In our study, the decoding of accuracy to distinguish between HS and LS declined, similar to previous decoding results based on spike information from ACC neurons^13^. However, in the chronic pain state, LFP-based decoding analyses provided distinction between HS and NS with very high accuracy. In fact, it is important to note that pain intensity decoding results from LFPs are quite similar to previous results based on spike activity of ACC population^13^. Therefore, we have reason to believe that LFP decoding may provide a robust strategy across a range of pain conditions. However, future studies are needed to examine the full spectrum of acute and chronic pain states, including nociceptive, inflammatory, and neuropathic pain.

In conclusion, we have found that LFPs in the ACC can provide important information regarding pain. Specifically, supervised machine learning can be used to analyze these LFP data to provide highly accurate prediction for the intensity and onset of pain. Our results support future studies that examine pain decoding using LFPs in various cortical and subcortical structures.

## Materials and Methods

### Animals

All procedures in this study were approved by the New York University School of Medicine (NYUSOM) Institutional Animal Care and Use Committee (IACUC) as consistent with the National Institute of Health (NIH) Guide for the Care and Use of Laboratory Animals to ensure minimal animal use and discomfort. Male Sprague-Dawley rats were purchased from Taconic Farms, Albany, NY and kept at Mispro Biotech Services Facility in the Alexandria Center for Life Science. Rats were kept with controlled humidity, temperature, and 12 hour (6:30 AM to 6:30 PM) light-dark cycle. Food and water were available ad libitum. Animals arrived to the animal facility at 250 to 300 grams and were given on average 10 days to adjust to the new environment prior to the onset of experiments.

### Experimental protocol and neurophysiological recordings

The tetrode microdrive (VersaDrive8, Neuralynx) assembly and implantation was similar to our previous study^13^. Tetrode was constructed using 12.7 µm nichrome wire(Sandvik) based on protocol^13,45^. Eight tetrodes were mounted in the VersaDrive8. Tetrode tip was cut using sharp scissor (Fine Science Tools) and then gold plated until the impedance was between 100 and 500 kΩ (NanoZ, Neuralynx). After that, the tetrode tips were soaked in the 100% Ethanol before implantation.

For implantation, rats were anesthetized with isoflurane (1.5–2%). A craniotomy was performed over unilateral anterior cingulate cortex (AP +2.5–3.5 mm, ML 0.8–1.8 mm). Tetrode bundle was lowered slowly at DV 1.6 mm with tip angel 10° toward the midline. Kwik-cast (World Precision Instruments) was used to seal the exposed area of the craniotomy. Ground and reference screws were anchored above cerebellum. Dental cement was used to secure the microdrive with bone screws. Rats were allowed to recover for about 1 week after surgery.

### Double laser stimulations and neural recordings

The double laser stimulation was performed as described previously^13^. Before stimulation, animals were allowed to habituate in a recording chamber over a mesh table for 30-min. Two diode-pumped solid-state lasers (SDL-473-1000T, Shanghai Dreams Laser Technology) were used to deliver noxious stimulation. The laser coupled patch cable fiber output intensity was calibrated (M83L01, Thorlabs) to one of three power intensities: 50 mW (NS), 150 mW (LS), and 250 mW (HS). And three stimulus condition pairs (NS vs LS, NS vs HS, LS vs HS) were used for each recording session. During each session, two lasers were randomly applied to rat’s hind paw for a total of approximately 60 trials with variable inter-trial intervals (approximately 1 min). The whole process was monitored by high speed camera (HC-V550, Panasonic). TTL pulses were used to synchronize with neural signals.

Tetrodes were lowered in steps of 120 µm before each day of recording. Raw neural signals were recorded with 32-ch digital headstage (RHD2132, Intan Technologies) and acquisition board (Open Ephys) at a sample rate of 30 kHz. To get spike activity, the raw data was band pass filtered from 300 Hz to 7.5 kHz and offline sorted by commercial software (Offline Sorter, Plexon). To get LFPs, raw data were digitally filtered by a bandpass filter between 0.3 and 300 Hz and down-sampled at 1 kHz.

### Data preprocessing

For multi-channel LFP signals, we first conducted a basic preprocessing procedure (detrend analysis, artifact channel rejection). We further performed principal component analysis (PCA) on the multi-channel LFP signals, and then projected the LFP signals onto the first dominant principal subspace. We extracted the denoised single-channel LFP signals for subsequent spectrum analyses.

### Spectrum analysis

We conducted spectrum analyses of LFP signals using a multitaper method (as opposed to the standard arbitrary windowing method). The multitaper method is an advanced spectral analysis technique^46^, which aims to reduce the bias/variance of spectral estimates by pre-multiplying the data with several orthogonal tapers known as Slepian functions. Specifically, we chose a half-bandwidth parameter *W* such that the windowing functions are maximally concentrated within [−*W, W*]. We chose *W* > 1/*T* (where *T* denotes temporal duration) such that the Slepian taper functions are well concentrated in frequency and have bias reducing characteristic.

The multitapered spectrum analysis was implemented with the Chronux toolbox^47^: an open source data analysis software at http://chronux.org. Spectrum and spectrogram were computed using functions ‘*mtspectrmc*’ and ‘*mtspecgramc*’, respectively. In terms of Chronux function setup, we used the tapers setup [*TW K*], where *TW* = 3 is the time-bandwidth product, and *K* = 2x*TW* *−* 1 = 5 is the number of tapers.

We used [0, 5] s data to compute the LFP power spectrum, where 0 denotes the laser onset. In addition, we compute the Z-score of power related to the baseline period [−5, 0] s right before the laser onset. A positive Z-score shows an increase in power at specific frequency band(s). The single band power was calculated by summing up all the estimated power values with each band (theta 4–8 Hz, alpha 8–15 Hz, beta 15–30 Hz, low gamma 30–60 Hz, high gamma 60–100 Hz).

### Spike, LFP, and Spike-LFP decoding

The goal of population-decoding analysis was to classify the trial labels of different stimulation intensities (e.g., LS vs. HS) based on spikes or LFP alone, or spikes and LFP combined. First, in spike alone decoding, for each single neuronal recording, we binned spikes into 50 ms to obtain spike count data in time and used a 50 ms moving window to accumulate spike count statistics from the laser onset (time 0) until 5 s (i.e., 100 bins). We assessed the decoding accuracy at each time bin based on the cumulative spike count statistics. Therefore, for a total of *C* neurons, the input feature dimensionality ranged from *C* (the first bin) to 100 *C* (all bins). Second, in single band LFP decoding, we used a 1-s window (with an incremental step size of 50 ms moving window) to compute the LFP power sum at each frequency band (theta, alpha, beta, low and high gamma). For all frequency band decoding, we combined all those 5 single band power at each bin together as feature vector. Finally, in spike plus LFP decoding, we combined the spike count and LFP power statistics as the input features.

In the experiments to classify different laser intensities (e.g., NS vs. HS, or LS vs HS), it was assumed that we have *n*_1_ trials under laser intensity 1, and *n*_2_ trials under laser intensity 2. We split the total (*n*_1_ + *n*_2_) trials into two groups, 80% used for training, and 20% used for testing. Specifically, we used a support vector machine (SVM) classifier^48^. The SVM is a discriminative supervised learning model that constructs the classification boundary by a separating hyperplane with the maximum margin. Specifically, the SVM can map the input **x**_*i*_ (*i* = 1, …, *N*, where *N* denotes the training sample size) into high-dimensional feature spaces that allows nonlinear classification.

1 $$y=\sum _{i=1}^{N}{\alpha }_{i}K({\bf{x}},\,{{\bf{x}}}_{i})+b$$

where *y*_*i*_ ∈ {−1, +1} denote the class label for the training sample **x**_*i*_ (some of which associated with nonzero *α*_*i*_ are called support vectors), *b* denotes the bias, and *K*(•,•) denotes the kernel function. We used a polynomial kernel and trained the nonlinear SVM with a sequential minimal optimization algorithm (MATLAB Machine Learning Toolbox: ‘*fitcsvm*’ function). Finally, the decoding accuracy was assessed by 5-fold cross-validation from 100 Monte Carlo simulations. We reported the mean ± S.E.M. decoding accuracy.

As a control, we also computed the chance-level decoding accuracy. We randomly permuted class labels between two classes and repeated the decoding analysis. This shuffling procedure was repeated 500 times, and we reported the chance level by the averaged classification accuracy based on shuffled data with permuted labels. In theory, when the sample sizes from both classes are perfectly balanced, the chance level should be close to 50%.

### Detecting the onset of acute pain

Using the moving window, we assessed the accumulative SVM decoding accuracy in time using spikes, LFP, or spike and LFP features together. At the onset of the laser stimulation, the decoding accuracy is around the chance level (~50%). To determine the onset of pain signals from the decoding perspective, we needed a threshold criterion from which we defined the time that crosses the threshold as the onset of pain signals. Here, we used the 1/*e* criterion (where e denotes the Euler’s number, and 1/*e* ≈ 0.37). The rationale of this choice was motivated by the criterion for defining the visual neuronal receptive field size^33^. We defined the onset of time as the first moment where the SVM decoding peak accuracy drops to the 1/*e* level of the dynamic range, where the dynamic range is defined as the difference between the peak accuracy and chancel level accuracy. Since the 1/*e* criterion is rather ad hoc, we also investigated whether changing the criterion (such as ½) would affect our results.

### Statistics and statistical tests

We reported the mean ± S.E.M. statistics in LFP power. Unless stated otherwise, we used the nonparametric Mann-Whitney rank-sum test for comparing the median statistics between two groups.

## Electronic supplementary material

Supplementary Figure
