# Supplementary material for: Local field potential decoding of the onset and intensity of acute pain in rats
Source: Sci Rep. 2018 May 29;8:8299. doi: 10.1038/s41598-018-26527-w (PMC5974270; doi:10.1038/s41598-018-26527-w)
Supplement: Supplementary file 1 — Supplementary Figure [file 41598_2018_26527_MOESM1_ESM.pdf]

Local field potential decoding of the onset and intensity of acute pain in rats

Qiaosheng Zhang[1, 5], Zhengdong Xiao[2, 3, 5], Conan Huang[1], Sile Hu[2, 3], Prathamesh Kulkarni[1, 3], Erik Martinez[1], Ai Phuong Tong[1], Arpan Garg[1], Haocheng Zhou[1], Zhe Chen[3,4, *], and Jing Wang[1, 4, *]

[1]Department of Anesthesiology, Perioperative Care, and Pain Medicine, New York University School of Medicine, New York, New York 10016. [2]College of Biomedical Engineering and Instrument Science, Zhejiang University, Hangzhou, Zhejiang, China. [3]Department of Psychiatry, New York University School of Medicine, New York, New York 10016. [4]Department of Neuroscience and Physiology, New York University School of Medicine, New York, New York 10016.

[5] These authors contributed equally.

Corresponding author *: correspondences should be addressed to Dr. Zhe Chen (zhe.chen3@nyumc.org) or Dr. Jing Wang (jing.wang2@nyumc.org), 450 East 29th Street, Room 823, New York, NY 10016.

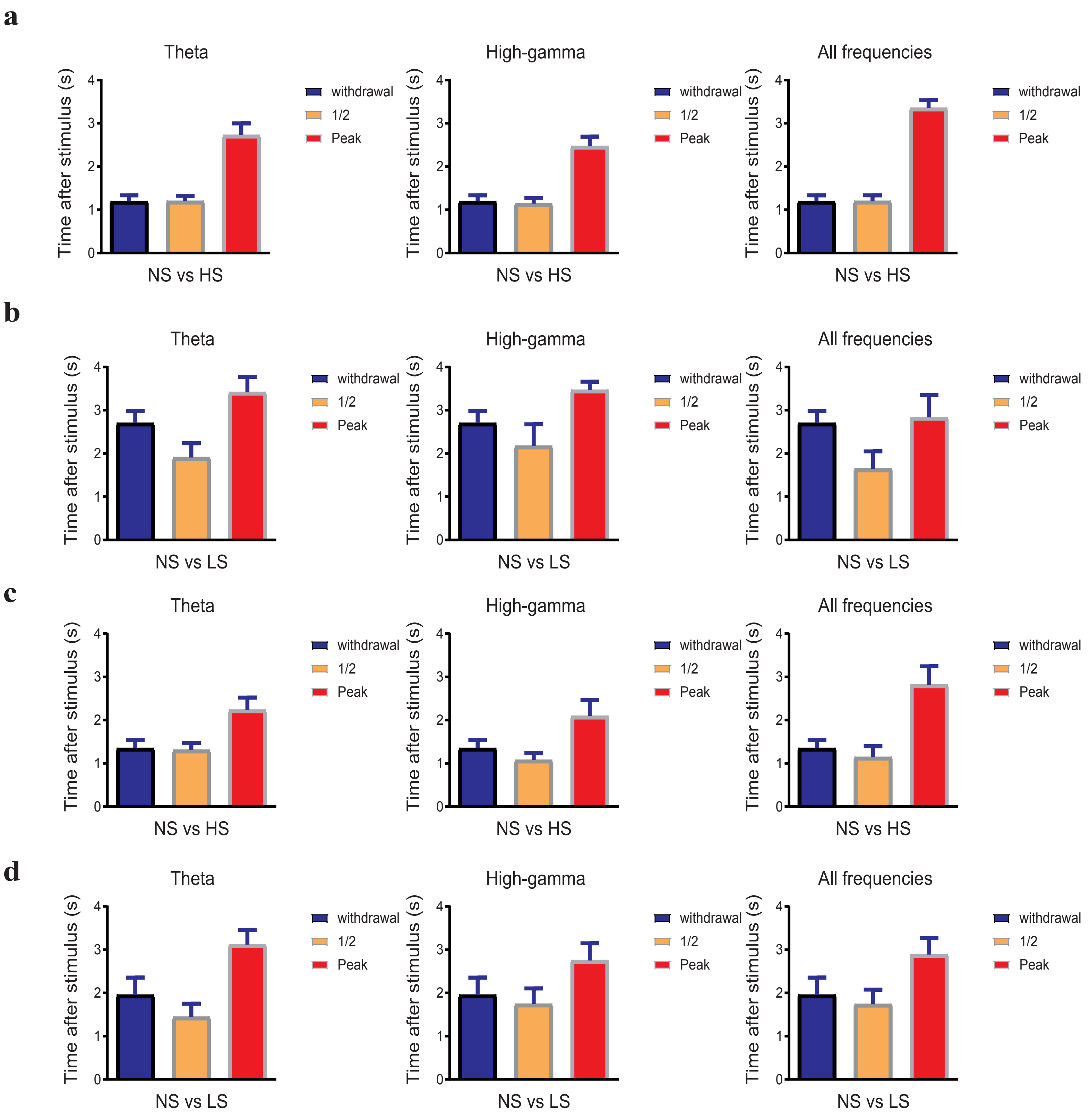
a
Theta
High-gamma
All frequencies
Time after stimulus (s)
0
1
2
3
4
withdrawal
1/2
Peak
NS vs HS
b
Theta
High-gamma
All frequencies
Time after stimulus (s)
withdrawal
1/2
Peak
NS vs LS
c
Theta
High-gamma
All frequencies
Time after stimulus (s)
withdrawal
1/2
Peak
NS vs HS
d
Theta
High-gamma
All frequencies
Time after stimulus (s)
withdrawal
1/2
Peak
NS vs LS

Supplementary Figure 1. **Using a 1/2 threshold to predict the onset of pain.** (**a**) Assessment of the onset for pain perception in response to HS is based on the time where the decoding accuracy (NS vs HS) reached the ½ of threshold, using LFP features from different frequency bands (from left to right: theta, high-gamma, all frequency). This assessment of pain perception corresponds well with latency to withdrawal from the stimulus onset. (**b**) Same analysis as panel **a**, but for NS vs. LS. (**c**) Same analysis as panel **a**, for CFA-treated rats. (**d**) Same analysis as panel **b**, for CFA-treated rats.
